# Efficacy of detergent-based cleaning methods against coronavirus MHV-A59 on porous and non-porous surfaces

**DOI:** 10.1080/15459624.2021.2015075

**Published:** 2022-01-28

**Authors:** Rachael L. Hardison, Sarah W. Nelson, Daniela Barriga, Jessica M. Ghere, Gabrielle A. Fenton, Ryan R. James, Michael J. Stewart, Sang Don Lee, M. Worth Calfee, Shawn P. Ryan, Megan W. Howard

**Affiliations:** a Battelle Memorial Institute, Columbus, Ohio;; b U.S. Environmental Protection Agency, Durham, North Carolina

**Keywords:** Cleaning, coronavirus, disinfection, fomites, high-touch surfaces, virucide, virus

## Abstract

This study evaluated the efficacy of detergent-based surface cleaning methods against Murine Hepatitis Virus A59 (MHV) as a surrogate coronavirus for SARS-CoV-2. MHV (5% soil load in culture medium or simulated saliva) was inoculated onto four different high-touch materials [stainless steel (SS), Acrylonitrile Butadiene Styrene plastic (ABS), Formica, seat fabric (SF)]. Immediately and 2-hr post-inoculation, coupons were cleaned (damp wipe wiping) with and without pretreatment with detergent solution or 375 ppm hard water. Results identified that physical removal (no pretreatment) removed >2.3 log_10_ MHV on ABS, SS, and Formica when surfaces were cleaned immediately. Pretreatment with detergent or hard water increased effectiveness over wet wiping 2-hr post-inoculation; pretreatment with detergent significantly increased (*p* ≤ 0.05) removal of MHV in simulated saliva, but not in culture media, over hard water pretreatment (Formica and ABS). Detergent and hard water cleaning methods were ineffective on SF under all conditions. Overall, efficacy of cleaning methods against coronaviruses are material- and matrix-dependent; pre-wetting surfaces with detergent solutions increased efficacy against coronavirus suspended in simulated saliva. This study provides data highlighting the importance of incorporating a pre-wetting step prior to detergent cleaning and can inform cleaning strategies to reducing coronavirus surface transmission.

## Introduction

The emergence of the severe acute respiratory syndrome coronavirus-2 (SARS-CoV-2), and the resulting 2019 coronavirus disease (COVID-19) pandemic, has highlighted the need for evidence-based guidelines to reduce viral transmission. While SARS-CoV-2 is now known to be primarily transmitted via respiratory droplet transmission (Kampf et al. 2020; CDC 2021), a proportion of SARS-CoV-2 infections may occur from surface transmission from contaminated objects and surfaces. While SARS-CoV-2 transmission from contaminated surfaces is thought to be low, some degree of transmission is through direct or indirect contact with contaminated surfaces (CDC 2021). This transmission depends on several factors: material type (porous vs. non-porous), surface stability of the virus, and infectious dose (Biryukov et al. 2020; Harvey et al. 2021; Kratzel et al. 2020;Chatterjee et al. 2021; Wilson et al. 2021). During the COVID-19 pandemic, numerous countermeasures, including routine cleaning and disinfection with an EPA-approved disinfectant, were recommended. More recently, updated guidance provided by CDC suggested routine cleaning without disinfection was sufficient for most circumstances.

Surface cleaning combines surfactant-based (e.g., detergents, soaps) or abrasive cleaners with physical removal to remove foreign material from surfaces (e.g., dirt, dust, or other organic debris [including microbes]); the physical removal (wiping or scrubbing) is a key component of this process. Residual non-microbial material (or soil) on surfaces can interfere with the antimicrobial activity of some chemical disinfectants by acting as a physical barrier or forming a chemical-soil complex with reduced antimicrobial activity (Lewis and Arens 1995; Muscarella 1995;Wyrzykowska-Ceradini et al. 2019). For this reason, disinfection strategies often incorporate surfactant-based cleaning prior to registered disinfection product application. Real-world cleaning methods vary by chosen product, but typically consist of wiping- or scrubbing- generated friction (by cloth, wipe, mop, or sponge) in addition to applying the cleaning solution.

In 2021, the Centers for Disease Control and Prevention (CDC) published updated guidelines for cleaning and disinfecting households and public spaces following the onset of the COVID-19 pandemic. The CDC guidelines state that, in most cases, cleaning high-touch surfaces with a household cleaner containing soap and water can reduce the risk of transmission of coronavirus from contaminated surfaces without the addition of a disinfectant (CDC 2021). While the CDC recommended chemical-based disinfectants when individuals in the homes (or community locations) were infected with COVID-19, routine cleaning with soap or detergent was recommended otherwise (CDC 2021).

Several studies show that physical cleaning methods can reduce over 90% of microbes on hard, non-porous surfaces, however these studies focused on bacterial pathogens or non-enveloped viruses (Gibson et al. 1999; Barker et al. 2004; Delhalle et al. 2020). Data on the susceptibility of SARS-CoV-2, or related enveloped viruses, remains limited. As an enveloped virus, SARS-CoV-2 is susceptible to some degree of membrane disruption or damage from surfactants in household cleaning products; 0.1% sodium laureth sulfate (SLS), a surfactant present in dishwashing liquid, can inactivate SARS-CoV-2 on hard, non-porous surfaces after a 30-sec contact time (Gerlach et al. 2020).

To assess the efficacy of cleaning methods against SARS-CoV-2, this study evaluated a surrogate coronavirus, Murine Hepatitis Virus A59 (MHV). MHV is an enveloped, positive-sense RNA *betacoronavirus* in the family *Coronaviridae*, like SARS-CoV-2. All *betacoronaviruses* contain similar virion structure, genome organization, and biophysical properties (Chen et al. 2020), however MHV is a risk group II pathogen, allowing experimental approaches to be performed in a biosafety level 2 (BSL-2) laboratory. Using MHV as a surrogate, therefore, reduced containment requirements, risk to staff, and costs.

This study evaluated the efficacy of cleaning measures including surfactant-based chemical and physical (wiping) methods on coronavirus-contaminated surfaces. We evaluated the efficacy of real-world cleaning methods to remove infectious coronaviruses from non-porous (stainless steel (SS), ABS plastic (ABS), Formica) and fibrous porous material (bus seat fabric [SF]). The purpose of this study was to evaluate the efficacy of cleaning with detergent-based solutions against coronaviruses on real-world materials.

## Materials and methods

### Cells and virus

MHV is a Risk Group II agent. All work was performed within a designated, limited access BSL-2 laboratory and all manipulation of MHV was performed within a class II Biosafety Cabinet (BSCII). Laboratory personnel always wore personal protective equipment (PPE; consisting of laboratory coats, double nitrile gloves, Tyvek sleeves, and safety glasses) while working with MHV. All laboratory personnel were trained on standard BSL-2 laboratory procedures and precautions prior to starting experiments.

MHV and murine L2 cells were kindly provided by Dr. Julian Leibowitz (Texas A&M College of Medicine, College Station, TX). MHV was propagated in 17 Clone 1 cells (17CL-1), a mouse fibroblast cell line (Sturman and Takemoto 1972), kindly provided by Dr. Susan Baker (Loyola University, Chicago, IL). 17CL-1 cells were incubated at 37 °C, 5% CO_2_ in Dulbecco’s Modified Eagle’s Medium with Glutamax (DMEM, Gibco, ThermoFisher Scientific, Allentown, PA), supplemented with 10% Fetal Bovine Serum (FBS, Omega Scientific), and 1% Penicillin-Streptomycin (P/S; ThermoFisher Scientific) in 75 cm^2^ tissu-eculture treated flasks (T-75, CellTreat, Pepperell, MA). MHV was propagated on 90% confluent 17Cl-1 cells at a multiplicity of infection (MOI) of 0.3 in low-FBS media (DMEM with Glutamax, 2% FBS, 1% P/S) with a 1-hr room temperature adsorption. Following adsorption, low-FBS media was added to each flask for a final volume of 12 mL per flask and incubated at 37 °C, 5% CO_2_. Virus was harvested at 80–90% cytopathic effect (CPE); flasks were frozen at −80 °C overnight, thawed at room temperature protected from light and clarified (2000 × *g*, 4 °C, 20 min). Clarified viral lysate was aliquoted and stored at −80 °C in single use (1 mL) vials for testing. MHV was titered on L2 cells. 50% Tissue Culture Infectivity Dose assays (TCID50) were performed on L2 cells seeded at 3 × 10^4^ cells per well in 96-well plates. L2 cells were incubated at 37 °C, 5% CO_2_ in DMEM, supplemented with 10% FBS, and 1% P/S. MHV for coupon inoculation was prepared in a 5% FBS soil load. Viral stocks were diluted 60:40 (volume: volume) in either DMEM (5% FBS) or simulated saliva (5% FBS). Simulated saliva was prepared as described previously (Woo et al. 2010) modified to include a ten-fold increase in phosphates (final concentration of 15 mM K_2_ HPO_4_, 24.6 mM K_2_HPO_4_).

### Coupons and cleaning products

Fatigue-Resistant 301 SS (SS; 0.03 cm thick; hardness rating of C40 on Rockwell Scale; meeting ASTM A666 specifications) and Impact-Resistant ABS (0.95 cm thick; hardness rating of R101-R109; meeting UL 94 HB specifications) was purchased from McMaster-Carr (Aurora, OH). Formica laminate was purchased from Home Depot (Columbus, Ohio). SF was sourced from American Seating (Item # 00333uw1; Grand Rapids, MI). All materials were cut into 7.62 cm × 1.91 cm (3 in × 0.75 in) coupons. Once cut, SS and ABS coupons were cleaned by soaking in a 1:100 diluted Liqui-Nox solution at pH 8.5 (Alconox, White Plains, NY), followed by rinsing in distilled water. Formica coupons were cleaned with 70% isopropanol and wiped with a cloth. All coupons were air-dried and packaged in heat-sealed polyethylene (PE) in packs of nine and sterilized by Electron Beam (EBEAM Services, Inc., Lebanon, OH) with a dose of 40kGy.

Detergent products, active ingredients, and method(s) of application tested in this study are listed in Table 1. Three cleaning procedures were evaluated for efficacy against surface-bound MHV in cell culture media or simulated saliva: (1) spray-application of detergent solutions followed by wiping with a hard water-dampened wipe, (2) spray-application of hard water followed by wiping with a hard water-dampened wipe, and (3) only wiping with a hard water-wetted wipe. For products that were diluted, 375 parts per million (ppm) hard water was prepared as described in US EPA SOP MB-30-02 (EPA 2019) and tested for hardness (acceptable range 338–394 ppm) using a Hach Model 5B Hardness Test Kit (Hach, Loveland, CO). Dawn Ultra Dishwashing Liquid (referred to as Dawn) and Tide Original Plus Bleach Alternative (Staples, Framingham, MA) were diluted in hard water to match concentrations for household use as suggested by each manufacturer per the product labels. The manufacturer recommended range for Dawn usage in a 37 L U.S. sink is 29–58 mL; this study used 43.5 mL Dawn per 37 L water for all testing. This study used 38.8 mL Tide Plus Bleach Alternative (referred to as Tide) per 75.7 L water (manufacturer’s recommended concentration).

### Application of cleaning products by trigger-pull sprayer

All detergent cleaning solutions were prepared new for each testing day. Prepared cleaning solution (250 mL) was added to a 32 oz ZEP Professional Sprayer Bottle (946.35 mL capacity; ZEP Inc., Atlanta, GA) set to the finest mist setting (1 mm nozzle opening). At each time point, triplicate coupons were removed from the humidity chamber (if applicable) and laid flat in a Class II BSC. The ZEP sprayer was held 15.24 cm to 20.32 cm (6–8 in.) above the coupons and passed lengthwise once in each direction over the coupons; during each pass the trigger was evenly and completely depressed to deposit the solution onto the coupons. Using the same methods, triplicate control coupons were sprayed with hard water. Coupons were wiped with a Kimwipe (Fisher Scientific, Allentown, PA) immediately after the spray-application of the cleaning solution or hard water only. Prior to wiping, Kimwipes (wipes) were folded into a 5.08 cm × 5.08 cm (2 × 2 in) square and dampened with 1.0 mL hard water; coupons were wiped lengthwise across the coupon once in each direction, ensuring that the wipe covered the entire coupon plus a 50% overlap on each pass. After wiping, coupons were extracted as described.

### Coupon extraction

Coupons were extracted in 5.0 mL low-FBS cell culture medium (DMEM, 2% FBS, 1% P/S) in a conical tube. Coupons were vortexed in extraction media for two min (Vortex-Genie 2, Scientific Industries, Inc., Bohemia, NY) on the maximum setting (~3200 rpm) using a 50 mL conical adapter (no more than five samples at a time), followed by inversion (three times). Extracts were passed through a Sephadex gelfiltration column (Sephadex G25 packed in PD-10 disposable columns, GE Healthcare Chicago, IL) via centrifugation (2 min, 1000 × g). The percent recovery of MHV inoculated onto coupons was greater than 93% across all materials when MHV was inoculated in culture medium (Supplementary material, Table S2), and greater than 88% when MHV was inoculated in simulated saliva (Supplementary material, Table S3).

### Cytotoxicity assays

The cleaning products and coupon materials were evaluated for cytotoxicity on L2 cells. Treated and untreated coupons of each material were generated as described; all coupons were inoculated with 0.1 mL of cell culture media (DMEM supplemented with 5% FBS). All coupons were extracted as described and cytotoxicity was assessed on L2 cells in 96-well tissue culture plates. A 0.1 mL aliquot of each extract was inoculated undiluted, or at 1:10 dilution into replicate (n = 8) wells of L2 cells at 80–85% confluency. Cells were maintained at 37 °C, 5% CO_2_ and assessed for cytotoxicity at 2 days post-inoculation (p.i.) via the CyQuant Lactate Dehydrogenase (LDH) Release Kit (Invitrogen, Cat# C20301; ThermoFisher Scientific, Waltham, MA). Cytotoxicity was evaluated using the following equation:

(1) $$\text{\%}\text{ Cytotoxicity }=\frac{\text{ treated LDH activity }-\text{ untreated LDH activity }}{\text{ Maximum LDH activity - untreated LDH activity }}\times 100$$

Results are reported as percent cytotoxicity relative to the average maximum possible cell death.

### Efficacy testing

Coupons (7.62 cm × 1.91 cm) were placed in individual sterile 10 cm petri dishes; triplicate coupons were inoculated with 0.1 mL MHV in DMEM, or simulated saliva supplemented with 5% FBS (i.e., 5% soil loading by volume) at an average titer of 2.23 × 10^6^ ± 8.36 × 10^5^ (virus in culture media) or 2.05 × 10^6^ ± 8.91 × 10^5^ (virus in saliva) TCID50 per coupon. Virus was inoculated in droplets evenly across the surface of the coupon in a straight line. Products were applied to coupons either immediately p.i. (T0 hr) or at 2 hr p.i. (T2). For coupons tested at T2, inoculated coupons were placed in a humidity chamber (23.05 ± 0.78% relative humidity) containing Drierite (W.A. Hammond Drierite Co., Xenia, OH) and held at room temperature (21.2 ± 1.1 °C) for 2 hr ± 5 min. During each test, untreated, inoculated, time-matched coupons were included in triplicate as controls.

### Quantification of infectious virus

MHV titer was determined by 50% TCID50 on L2 cells infected at 80–90% confluency in 96-well plates. Briefly, extracts were serially diluted in low-FBS containing media (DMEM supplemented with 2% FBS) and 0.1 mL of each neat or diluted sample was plated onto replicate wells (n = 12) and incubated for 2 days at 37 °C and 5% CO_2_; plates were scored for cytopathic effect (CPE) 2 days post-infection. Titer was determined via the Reed-Muench method (Reed and Muench 1938). Assay limit of detection (LOD) was dependent on the lowest readable dilution: LOD was 10 TCID50 per mL (1.0 log_10_ TCID50 per mL) for undiluted samples and 100 TCID50 per mL (2.0 log_10_ TCID50 per mL) when the lowest readable dilution was 1:10.

### Calculations and statistical analysis

Percent reduction, log reduction, and pooled error for log reduction, where appropriate, were calculated using Equations 2–4. For each cleaning product, a dynamic range (defined as the window of infectivity able to be observed) was calculated for each test using Equation 5 below:

(2) $$\text{\% Reduction }=\frac{\left(\text{ Untreated Titer })-(\text{ Treated Sample Titer }\right)}{\left(\text{ Untreated Titer }\right)}\times 100$$

(3) $$\text{Log Reduction }={\text{log}}_{10}(\text{ Untreated titer })-{\text{log}}_{10}(\text{ Treated Sample titer })$$

(4) $$\text{ Pooled Error }={\left[\left(\frac{\text{StDev}{\left(\text{log}\frac{\text{TCID}50}{mL}\text{ untreated coupons }\right)}^{2}}{3}\right)+\left(\frac{\text{StDev}{\left(\text{log}\frac{\text{TCID}50}{mL}\text{ treated coupons }\right)}^{2}}{3}\right)\right]}^{0.5}$$

(5) $$\text{ Dynamic Range for Chemical }={\text{log}}_{10}(\text{ Untreated titer })-{\text{log}}_{10}\text{LOD}$$

Pursuant to the Environmental Protection Agency’s (EPA) “Product Performance Test Guidelines OCSPP 810.2200: Disinfectants for Use on Environmental Surfaces – Guidance for Efficacy Testing” (EPA 712-C-17-004) a product was considered efficacious against MHV if a 99.9%, 3-log reduction, was demonstrated for the product compared to untreated, time-matched coupons. It should be noted that the detergents tested are not EPA registered disinfectants; reference to the disinfection guidance is for comparison only. All test samples were performed in triplicate. Statistical significance was determined by ANOVA where applicable and p-value lower than 0.05 was considered statistically significant.

## Results

### MHV-A59 stability on coupon materials

**Figure 1.**
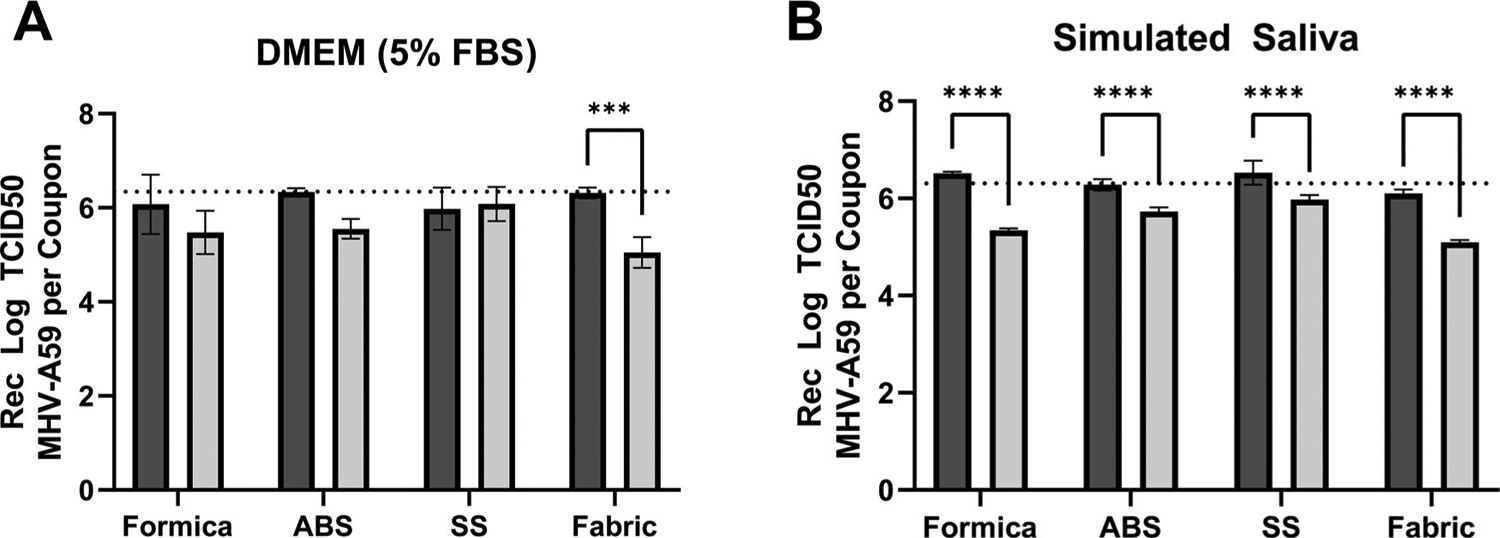
Surface stability of MHV-A59 in cell culture media or simulated saliva. Infectious virus (TCID50) in cell culture media (A) or simulated saliva (B) recovered at T = 0 (black bars) or T = 2 (gray bars) from Formica (n = 5), ABS plastic (ABS; n = 3), SS (SS; n = 5), or SF (Fabric; n = 5) coupons. Average inoculum titer per 0.1mL inoculated on each coupon (6.34 log10 TCID50 virus in DMEM, panel A; 6.31 log10 TCID50 virus in simulated saliva, panel B) is indicated by a dashed line. Displayed is the mean and standard deviation. Statistical significance was determined by 2-way ANOVA with Sidak’s multiple comparisons test. ***, *p* ≤ 0.001, ****, *p* ≤ 0.0001.

Stability of MHV on each coupon material at 2 hr p.i. was determined prior to efficacy testing. Viral suspensions of MHV in either cell culture media or simulated saliva (both containing a 5% soil load) were extracted from the coupon surface immediately (T0 hr) or 2 hours (T2 hr) after inoculation and quantified by TCID50. It should be noted that none of the coupon materials were cytotoxic to L2 cells (Supplementary material, Table S1). When virus was inoculated in cell culture media, recovery at T0 hr was >93% across all materials (Supplementary material, Table S2). There was no significant difference in the T0 hr or T2 hr recovery of MHV suspended in cell culture medium from Formica, ABS, or SS coupons; however, significantly less virus was recovered from the porous SF coupon material after the virus had been allowed to dry (T2 hr) when compared to immediate (T0 hr) recovery (*p* = 0.0010; Figure 1A, Supplementary material, Table S2). When virus was inoculated in simulated saliva, recovery at T0 was 82.4% from ABS plastic, and ≥100% from all other materials (Supplementary material, Table S3). Significantly less virus in simulated saliva was recovered from all coupon materials after 2 hr compared to coupons that were extracted immediately (*p* < 0.0001 for all materials; Figure 1B; Supplementary material, Table S2).

Overall, for both inoculum types (cell culture media or simulated saliva), extraction methods provided >82% virus recovery from all coupon surfaces regardless of virus matrix at T0 hr. Additionally, infectious MHV was stable across 2 hr on each coupon material, with limited loss of titer at T2 hr, however MHV stability may be dependent upon both matrix (saliva or cell culture media) and surface material.

### Efficacy of detergent-based cleaning methods against coronavirus in cell culture media

**Figure 2.**
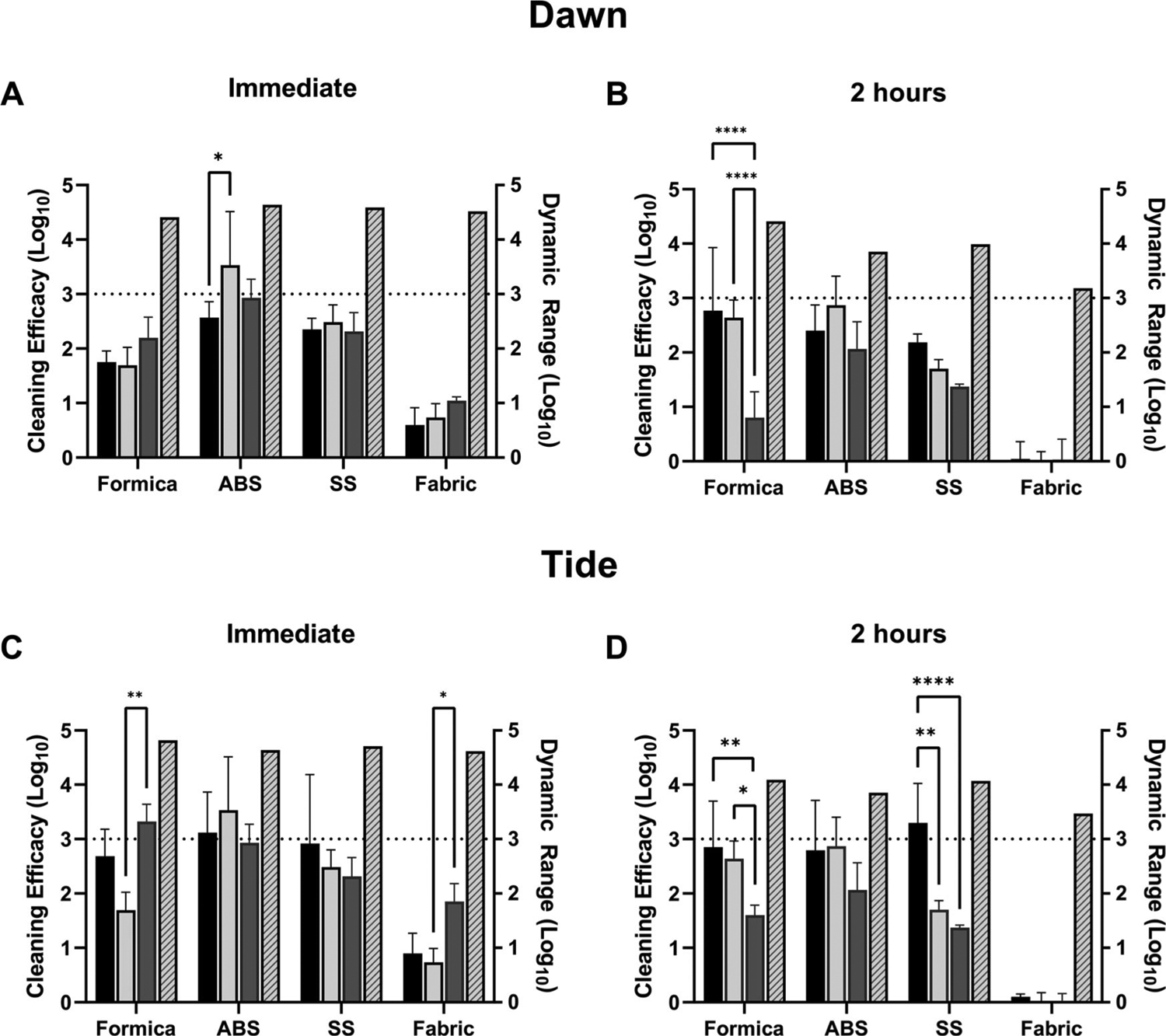
Efficacy of cleaning methods against MHV-A59 in 5% soil load in cell culture media. Cleaning method efficacy (Log_10_ reduction; solid bars, left-y axis) and dynamic range (Log_10_; patterned bars; right y-axis) across all coupon materials immediately p.i. (A, C) or 2-hr (B, D) p.i. of virus in cell culture media, 5% FBS onto the surface. Black bars, coupons pre-wetted with a solution containing Dawn Ultra (A, B) or Tide Plus Bleach Alternative (C, D) and wiped with a damp wipe. Light gray bars, coupons pre-wetted with hard water by trigger-pull sprayer and wiped with a water-dampened wipe. Dark gray bars, coupons wiped with a water-dampened wipe only (no pre-wetting step). Displayed is the mean and standard deviation. The target 3-log reduction is indicated by a dashed line on the y-axis. Statistical significance was determined by 2-way ANOVA with Tukey’s multiple comparisons test. *, *p* ≤ 0.05; **, *p* ≤ 0.01; ***, *p* ≤ 0.001; ****, *p* ≤ 0.0001.

Efficacy of Dawn and Tide was evaluated using trigger-pull spray application followed by wiping the coupon surface with a water-dampened wipe (Figure 2, Supplementary material, Table S4). This was compared to spraying with hard water followed by wiping with a dampened wipe and to only wiping with a dampened wipe. Both Dawn and Tide were non-cytotoxic (<10% cytotoxicity) to L2 cells (Table 2). At T0, virus was easily removed from hard, non-porous coupon surfaces (SS, ABS or Formica) by the physical action of wiping (water-dampened wipe only, Figure 2A,C) and the addition of detergent solution or with hard water did not significantly increase virus removal (Dawn, Tide, Figure 2A,C). At T0, reduction of MHV on Formica and SS ranged between an average of 1.75 and 2.35 log, irrespective of the cleaning method for Dawn detergent (Figure 2A). The spray with the Dawn solution or hard water prior to wiping with the dampened wipe was not statistically significantly different than wiping alone with the hard water-dampened wipe. Reduction in MHV was greatest for ABS; a greater than 3 log reduction was observed for the coupons sprayed with hard water prior to wiping. This result was not statistically significantly different from wiping alone, however, it was statistically different than for spraying with the Dawn detergent. Reduction in MHV was lowest for the SF, resulting in less than 1 log reduction with no differences among the cleaning methods (detergent spray/wipe, hard water spray/wipe, wipe alone).

Consistent with the results discussed above, cleaning with Tide resulted in a > 2.5 log reduction on all non-porous materials at T0; however, the addition of Tide did not significantly increase removal over spraying with hard water followed by wiping or by wiping the coupons with a damp wipe alone (Figure 2C). On SF, cleaning only resulted in a < 1 log reduction.

In contrast to results obtained at T0 hr, wetting agents (detergent or hard water) were more effective at removal of virus dried on Formica and SS surfaces at T2 hr. Spraying with hard water, Dawn solution, or Tide solution prior to wiping removed significantly more virus compared to wiping alone on Formica (Figure 2B, Dawn, 2.76 log reduction, *p* < 0.0001 vs. 0.80 log reduction with wiping alone; Figure 2D, Tide, 2.85 log reduction with Tide, *p* = 0.008 vs. 1.60 log reduction with wiping alone). However, virus removal by spraying with the Dawn or Tide solutions was not significantly different from removal by hard water (without added detergent) on Formica or SS (Figure 2B,D). The only condition where detergent removed significantly more virus when compared to hard water only was with the Tide solution on SS at T2 hr (Figure 2D, 3.29 log reduction with Tide, *p* = 0.0025 vs. 1.7 log reduction with hard water only).

These results suggest that removal of dried virus (T2, cell culture media, 5% soil load) from hard, nonporous surfaces is more effective when the surface is wetted with a wetting agent (hard water or detergent) prior to physical wiping. In general, cleaning of virus in cell culture media with detergent-based cleaning solutions does not appear to provide a significant benefit compared to wetting the surface with water alone. It should be noted that these coupons were pre-cleaned; cleaning soiled surfaces may benefit from the detergent-based cleaning method (a condition not tested in this current study).

### Cleaning method efficacy against virus in simulated saliva

**Figure 3:**
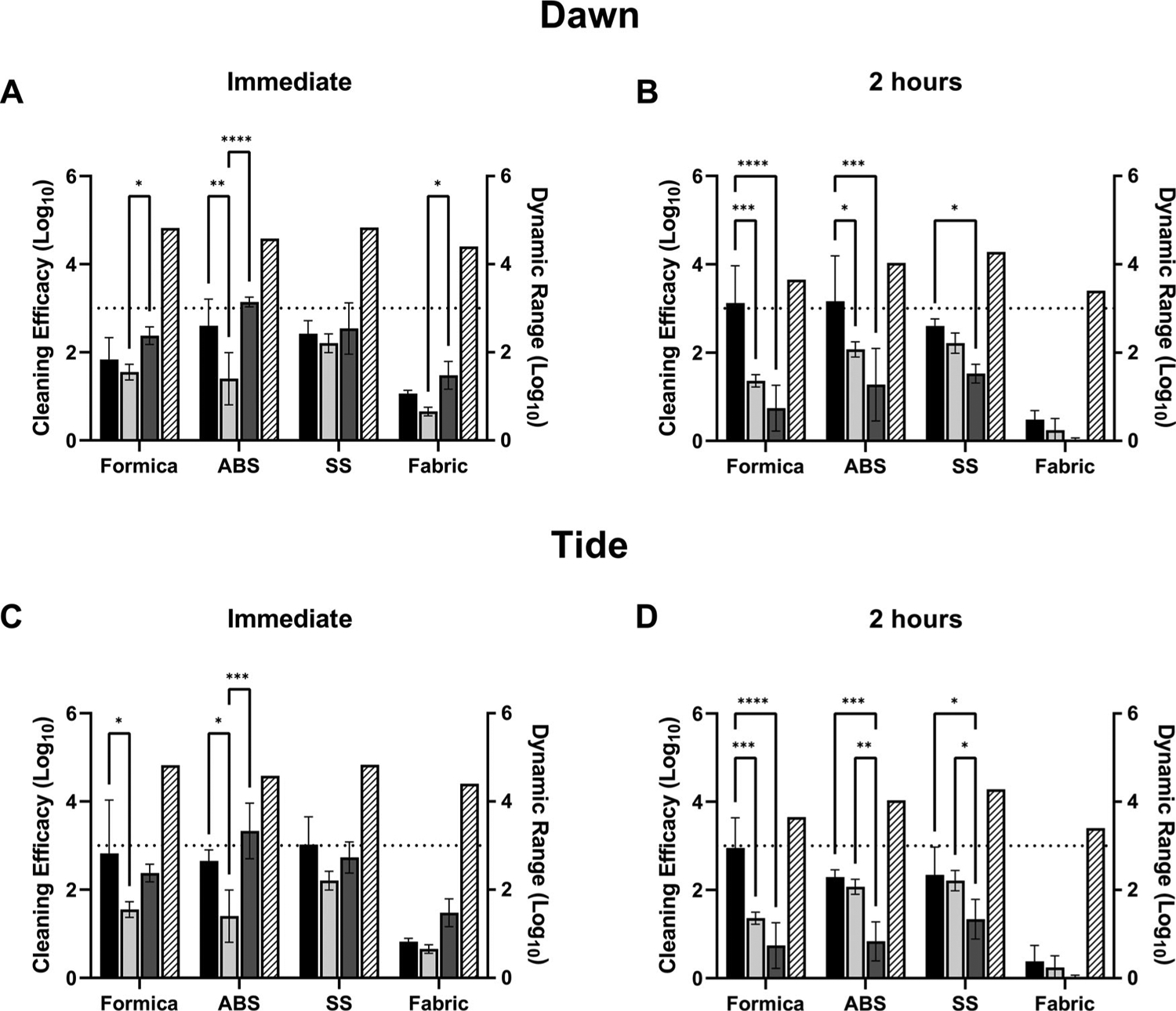
Efficacy of cleaning methods against MHV-A59 in simulated saliva. Cleaning method efficacy (Log_10_ reduction; solid bars, left-y axis) and dynamic range (Log_10_; patterned bars; right y-axis) across all coupon materials immediately p.i. (A, C) or 2-hours (B, D) p.i. of MHV in simulated saliva onto the surface. Black bars, coupons pre-wetted with a solution containing Dawn Ultra (A, B) or Tide Plus Bleach Alternative (C, D) and wiped with a water-dampened wipe. Light gray bars, coupons pre-wetted with hard water by trigger-pull sprayer and wiped with a damp wipe. Dark gray bars, coupons wiped with a water-dampened wipe only (no pre-wetting step). Displayed is the mean and standard deviation. The target 3-log reduction is indicated by a dashed line on the y-axis. Statistical significance was determined by 2-way ANOVA with Tukey’s multiple comparisons test. *, *p* ≤0.05; **, *p* ≤ 0.01; *** *p* ≤ 0.001; ****, *p* ≤ 0.0001.

Cleaning methods were also evaluated for efficacy against MHV suspended in simulated saliva (Figure 3; Supplementary material, Table S5). At T0 hr on SS, surface wiping with a dampened wipe (wipe alone) or spraying with hard water and then wiping with a dampened wipe had consistent efficacy results (log reduction) with cleaning by spraying with Dawn or Tide solutions followed by wiping with a dampened cloth (Figure 3A,C). On ABS, Dawn or Tide resulted in statistically increased removal over spraying with hard water followed by wiping (Figure 3A, Dawn, 2.60 log reduction, *p* = 0.0015 vs. 1.4 log reduction with hard water; Figure 3C, Tide, 2.65 log reduction, *p* = 0.0145 vs. 1.4 log reduction with hard water), however no statistical difference between Dawn or Tide treatments and wiping alone was observed. On Formica, the addition of Dawn did not significantly increase virus removal over hard water or wiping alone tests (Figure 3A; Dawn, 1.84 log reduction; *p* = 0.613 vs. hard water; *p* = 0.193 vs. wipe alone), however the addition of Tide did significantly increase (*p* = 0.0129) virus removal over hard water alone (Figure 3C; 2.82 log reduction with Tide vs. 1.55 log reduction with hard water). On porous SF, all methods (with or without a wetting agent) only resulted in <1.5 log reduction from untreated coupons (Figure 3A,C).

Tests were also performed at T2 hr to evaluate cleaning method efficacy against dried virus suspended in simulated saliva. At T2 hr, Dawn cleaning was significantly more effective on Formica and ABS over hard water or wiping alone (Figure 3B, Formica: *p* = 0.0007 vs. hard water, *p* < 0.0001 vs. wipe alone; ABS: *p* = 0.0353 vs. hard water, *p* = 0.0003 vs. wipe alone). Tide cleaning was also significantly more effective than hard water or wiping alone on Formica and ABS (Figure 3D; Formica, 2.95 log reduction with Tide, *p* = 0.0001 vs. 1.36 log reduction from hard water and *p* < 0.0001 vs. 0.74 log reduction with wipe only; ABS: 2.29 log reduction with Tide, *p* = 0.0004 vs. 0.83 log reduction with wipe only); however, there was no significant difference between Tide cleaning over hard water alone on ABS (Figure 3D). Virus removal from SS was variable. For both Dawn and Tide, no significant increase over hard water alone was observed, however both detergents removed significantly more virus than wiping alone (Figure 3B: Dawn: *p* = 0.0372 vs. wipe alone; Figure 3D: Tide: *p* = 0.0132 vs. wipe alone). No methods tested were effective at removing virus in simulated saliva from SF (Figure 3B,D).

On all hard, non-porous surfaces (SS and ABS), the application of a wetting agent to the coupon surface just prior to wiping increased removal of virus suspended in simulated saliva at 2 hr post-inoculation. These results suggest that the use of a detergent increases the effectiveness of cleaning methods to remove dried virus in saliva.

## Discussion

Throughout the COVID-19 pandemic, guidelines for cleaning and disinfection of households and public spaces often focused on disinfection methods. Recent updates to CDC guidelines suggest that cleaning methods (i.e., using soap and water to physically remove viral particles from a surface) are often sufficient to reduce the risk of transmission in households or public spaces even without the use of a disinfectant (CDC 2021). However, there is limited evidence on the effectiveness of cleaning methods against coronaviruses on surfaces. We evaluated the efficacy of household detergent-based cleaning methods against MHV on high-touch materials to determine the efficacy of real-world cleaning practices against coronaviruses. This work used a surrogate agent (MHV-A59), which, due to similar biophysical properties and genetic relatedness, allows insights into the efficacy of these methods against SARS-CoV-2.

In all tests, cleaning of soft fibrous porous material (SF) was ineffective regardless of whether a detergent solution was used. This was anticipated, given that the three-dimensional nature of porous materials increases the complexity of virus-surface interactions (Yeargin et al. 2016), and likely provides protection from physical removal and surfactant-based inactivation methods. However, it is also likely that viral binding to fibrous materials, such as seat fabric, presents a lower risk of indirect viral transmission compared to nonporous surfaces in real-world situations (Lopez et al. 2013). On harder surfaces (ABS, SS, and Formica) physical removal (wipe-only, wiping the coupon surface) did not consistently reduce MHV at ≥3-log reduction at T2 hr; the addition of detergent (Tide or Dawn) did substantially increase virus removal on Formica (Dawn, 3.12 log reduction; and Tide, 2.95 log reduction) for MHV in simulated saliva and SS (Tide; 3.29 log reduction) for MHV in culture media (Figures 2B,D and 3B,D). Results on SS were more varied for MHV in culture media, with no difference observed for both detergents at T0 hr (Figure 2A,C), no difference for Dawn at T2 hr (Figure 2B), but a significant increase in efficacy was observed for Tide at T2 hr (Figure 2D) over wiping alone. For MHV in simulated saliva, both Dawn and Tide showed significant increases over wiping alone at T2 hr (Figure 3B,D, ABS, SS, and Formica). Overall, MHV in simulated saliva was more consistently (and significantly) removed by cleaning methods than MHV in culture media at immediate and T2 hr suggesting that the virus matrix affects the ability of virus to be physically removed from surfaces via cleaning methods.

Our results show that MHV was removed from non-porous coupon surfaces by a water-dampened wipe (wipe-only, Figures 2 and 3, ABS, SS, and Formica), and in most cases removal by spraying first with hard water was as effective as spraying first with Dawn or Tide solutions prior to wiping (Figures 2 and 3). While wiping with water alone (either pre-wetting or wet wiping) was effective immediately after MHV is deposited on surfaces, detergent cleaning showed increased removal once the inoculum had dried. Physical removal with a water-dampened wipe (wipe-only) or spraying with hard water only was not surprising—previous efforts evaluated water-dampened cloth wiping methods and showed an average of 3.15 log_10_ norovirus removed (Gibson et al. 2012), similar to the removal observed in this study by wiping SS with the dampened wipe immediately p.i. (Supplementary material, Table S4; SS, culture media, 2.31 ± 0.35; Supplementary material, Table S5, SS, saliva, 2.64 ± 0.44).

In real-world situations, respiratory droplets from potentially infectious individuals may not be cleaned immediately after deposition on a surface. Our results show that although wiping with a damp wipe can be as effective as detergent based cleaning for wet virus, it might not be sufficient to remove dried virus from surfaces. Pre-wetting the surface with water or a detergent-based cleaning solution enhanced the removal of dried virus. Interestingly, matrix-specific differences were observed between water- and detergent-based cleanings. For MHV in culture media, detergent- and water-based cleaning was equally as effective (Figure 2, T0 hr and T2 hr). However, for MHV in simulated saliva and dried (T2 hr), detergent-based cleaning showed increased efficacy on SS, Formica, and ABS over spraying with water-only followed by wet-wiping. Formica and ABS required detergent-based cleaning to reach a 3-log reduction for MHV in simulated saliva (Figure 3).

Matrix differences may be explained by MHV binding to mucins in saliva. Coronaviruses bind mucins or mucin-like proteins (Schwegmann-Wessels et al. 2003), suggesting that virion-mucin complexes form in saliva. These complexes may enable stronger surface binding than virus in culture media. As mucinsurface adsorption is not achieved by dilution alone (Dėdinaitė and Bastardo 2002), surfactant-based cleaning likely provides additional desorption over wateralone. Detergents (like those in Dawn and Tide) remove pre-adsorbed mucin from surfaces through the formation of mucin-surfactant complexes or competitive adsorption (Dėdinaitė and Bastardo 2002), which may be the process by which coronavirus in saliva is removed more efficiently from surfaces during detergent-cleaning.

Our findings show that Dawn and Tide cleaning efficacy is material- and matrix-dependent, which aligns with reports that surfactant-based cleaning methods do not provide consistent surface-inactivation of viruses. For example, Tide laundry detergent (without bleach or a bleach alternative) does not provide consistent inactivation of the avian influenza virus on hard, non-porous surfaces (Alphin et al. 2009), and regular daily cleaning with nonionic and anionic surfactants was not sufficient to eradicate human coronavirus 229E from classroom surfaces (Bonny et al. 2018).

Surfactants, such as sodium lauryl sulfate in both Dawn and Tide, are known protein-denaturing agents (Pedersen et al. 2020), and are commonly thought to denature or induce conformational changes in viral surface proteins, resulting in loss of viral infectivity. However, our study did not extract virus directly from wipes, thus we are unable to determine if the methods inactivated virus, or only removed MHV from coupon surfaces.

## Conclusions

In summary, our study provides evidence that detergent-based cleaning methods can be effective against coronaviruses when used on high-touch, non-porous surface materials. As MHV is more stable than SARSCoV-2 on SS surfaces (Forni et al. 2017; Gorbalenya et al. 2020), we speculate that the cleaning method efficacies reported here may represent a conservative estimate of how effective these methods may be against SARS-CoV-2. Our results can be extrapolated from MHV to SARS-CoV-2 and support the CDC recommendations that detergent- and water-only cleaning methods can reduce SARS-CoV-2 on hard non-porous surfaces (ABS, SS, Formica). However, the results suggest these cleaning methods are much less effective on fibrous porous surfaces (SF). Additional studies are needed to confirm the efficacy of detergent and water cleaning methods against SARS-CoV-2, and a comparison of detergent and water cleaning methods with chemical disinfectants would be useful information to support infection control and risk reduction methods for surface-transmission.

## Supplementary Material

Supplementary Material

## Figures and Tables

**Table 1.** Cleaning products and application methods.

Product	Manufacturer	Dilution or preparation	Active ingredient(s)	Method of application
Dawn Ultra Dishwashing Liquid, Original Scent	Proctor & Gamble	Diluted in Hard Water	Sodium lauryl sulfate, sodium laureth sulfate, C10–16 alkyldimethylamine oxide, C9–11 Pareth-8, PEI-14 PEG-24/PPG-16 CoPolymer	Trigger-Pull Sprayer
Tide Plus Bleach Alternative, Original Scent	Proctor & Gamble	Diluted in Hard Water	Sodium and MEA Laureth Sulfate, Sodium and MEA C10–16 Alkylbenzenesulfonate, Sodium and MEA Laureth Sulfate, C10–16 alkyldimethylamine oxide, C10–16 Pareth,	Trigger-Pull Sprayer

**Table 2.** Cytotoxicity of cleaning solutions.

Product	Plate format	Dilution	Percent cytotoxicity	Stdev	Lowest readable dilution	Assay LOD (log_10_ TCID50 per mL)
Dawn Ultra	96	Neat	−1.40	4.92	Neat	1.0
Tide Plus Bleach Alternative	96	Neat	−0.01	9.62	Neat	1.0
375 ppm Hard Water	96	Neat	−0.02	5.26	Neat	1.0

## Data Availability

All data are publicly available at data.gov.
