# Supplementary material for: Efficacy of detergent-based cleaning methods against coronavirus MHV-A59 on porous and non-porous surfaces
Source: J Occup Environ Hyg. Author manuscript; Available in PMC 2023 Feb 1. (PMC8965596; doi:10.1080/15459624.2021.2015075)
Supplement: Supplementary Material [file NIHMS1788369-supplement-Supplementary_Material.pdf]

Efficacy of Detergent-Based Cleaning Methods Against Coronavirus MHV-A59 on Porous and Non-Porous Surfaces – Supplemental Materials

Table S1. Cytotoxicity of Coupon Materials.

Material	Average Percent Cytotoxicity (%) [a]	Stdev
**Formica**	3.20	7.14
**ABS**	0.47	2.40
**SS**	-0.93	1.32
**Fabric**	1.04	4.88

[a] Percent cytotoxicity relative to maximum LDH release in lysed control wells.

Efficacy of Detergent-Based Cleaning Methods Against Coronavirus MHV-A59 on Porous and Non-Porous Surfaces – Supplemental Materials

Table S2. Recovery of MHV-A59 in Culture Medium

Material	Inoculum Volume per Coupon (mL)	Recovery (Log_{10} TCID50 per Coupon)				Log Reduction from T0	Adjusted P Value [a] (T2 vs. T0)	Percent Inoculum Recovered at T0 (%)
		T0		T2				
		Avg	*Stdev*	*Avg*	*Stdev*			
**Formica**	0.1	6.07	0.63	5.48	0.46	0.60	0.115	95.30
**ABS**	0.1	6.33	0.07	5.55	0.21	0.78	0.103	99.37
**SS**	0.1	5.98	0.45	6.08	0.36	-0.10	0.992	93.87
**Fabric**	0.1	6.32	0.11	5.05	0.33	1.27	0.001	98.43

[a] Two-way ANOVA, Sidak's Multiple Comparisons Test

Efficacy of Detergent-Based Cleaning Methods Against Coronavirus MHV-A59 on Porous and Non-Porous Surfaces – Supplemental Materials

Table S3. Recovery of MHV-A59 in Simulated Saliva

Material	Inoculum Volume per Coupon (mL)	Recovery (Log_{10} TCID50 per Coupon)				Log Reduction from T0	Adjusted P Value [a] (T2 vs. T0)	Percent Inoculum Recovered at T0 (%) [b]
		T0		T2				
		Avg	*Stdev*	*Avg*	*Stdev*			
**Formica**	0.1	6.52	0.03	5.34	0.04	1.18	<0.0001	119.10
**ABS**	0.1	6.28	0.12	5.73	0.09	0.55	<0.0001	82.42
**SS**	0.1	6.53	0.25	5.98	0.09	0.55	<0.0001	158.00
**Fabric**	0.1	6.10	0.08	5.09	0.05	1.01	<0.0001	170.45

[a] Two-way ANOVA, Sidak's Multiple Comparisons Test; [b] Average percent recovery from calculated TCID50 value inoculum per coupon; n=3 per test.

Efficacy of Detergent-Based Cleaning Methods Against Coronavirus MHV-A59 on Porous and Non-Porous Surfaces – Supplemental Materials

Table S4. Cleaning Efficacy of Dawn and Tide Plus Bleach Alternative against MHV-A59 in Cell Culture Media on Material Coupons[a]

	Dawn		Tide		Hard Water		Wipe Alone	
	T0 hr	T2 hr	T0 hr	T2 hr	T0 hr	T2 hr	T0 hr	T2 hr
Formica	1.75 ± 0.21 (4.41)	2.77 ± 1.16 (4.41)	2.68 ± 0.50 (4.82)	2.85 ± 0.85 (4.09)	1.69 ± 0.33	2.64 ± 0.32	2.76 ± 0.69	1.20 ± 0.54
ABS	2.57 ± 0.29 (4.64)	2.40 ± 0.47 (3.85)	3.12 ± 0.75 (4.64)	2.79 ± 0.92 (3.85)	3.53 ± 0.99	2.87 ± 0.53	2.93 ± 0.31	2.06 ± 0.45
SS	2.35 ± 0.21 (4.59)	2.18 ± 0.16 (3.99)	2.92 ± 1.27 (4.71)	3.30 ± 0.72 (4.07)	2.49 ± 0.32	1.70 ± 0.17	2.31 ± 0.35	1.37 ± 0.04
Fabric	0.60 ± 0.32 (4.52)	0.04 ± 0.32 (3.18)	0.90 ± 0.37 (4.62)	0.10 ± 0.05 (3.47)	0.73 ± 0.26	0.002 ± 0.17	1.45 ± 0.49	0.03 ± 0.25

[a] The dynamic range for each test is included in parentheses. Data are presented as mean $\log_{10}$ reduction ± $\log_{10}$ standard deviation. The dynamic range ($\log_{10}$) is indicated in parentheses for test samples (detergents). For hard water and wipe alone controls, data reflects the mean and standard deviation values across both Dawn and Tide tests.

Efficacy of Detergent-Based Cleaning Methods Against Coronavirus MHV-A59 on Porous and Non-Porous Surfaces – Supplemental Materials

Table S5. Cleaning Efficacy of Dawn and Tide with Bleach Alternative against MHV-A59 in Simulated Saliva on Material Coupons [a]

	Dawn		Tide		Hard Water		Wipe Alone	
	0 hr	2 hr	0 hr	2 hr	0 hr	2 hr	0 hr	2 hr
Formica	1.84 ± 0.50 (4.82)	3.12 ± 0.84 (3.65)	2.82 ± 1.21 (4.82)	2.95 ± 0.68 (3.65)	1.55 ± 0.18	1.36 ± 0.14	2.38 ± 0.20	0.74 ± 0.52
ABS	2.60 ± 0.61 (4.58)	3.16 ± 1.03 (4.03)	2.65 ± 0.65 (4.58)	2.29 ± 0.17 (4.03)	1.40 ± 0.59	2.07 ± 0.17	3.24 ± 0.42	1.06 ± 0.64
SS	2.42 ± 0.30 (4.83)	2.60 ± 0.16 (4.28)	3.02 ± 0.63 (4.83)	2.34 ± 0.63 (4.28)	2.21 ± 0.21	2.21 ± 0.23	2.64 ± 0.44	1.43 ± 0.33
Fabric	1.06 ± 0.08 (4.40)	0.48 ± 0.21 (3.40)	0.82 ± 0.08 (4.40)	0.38 ± 0.36 (3.40)	0.66 ± 0.10	0.24 ± 0.27	1.48 ± 0.32	-0.09 ± 0.16

[a] The dynamic range for each test is included in parentheses. Data are presented as mean $\log_{10}$ reduction ± $\log_{10}$ standard deviation. The dynamic range ($\log_{10}$) is indicated in parentheses for test samples (detergents). For hard water and wipe alone controls, data reflects the mean and standard deviation values across both Dawn and Tide tests.
